# Bacteriological Study of Diabetic Foot Infections in an Iranian Hospital

**Published:** 2011-08-01

**Authors:** A Dezfulian, M T Salehian, V Amini, H Dabiri, M Azimi Rad, M M Aslani, M Alebouyeh, I Fazel, M R Zali

**Affiliations:** 1 Research Center for Foodborne and Diarrheal Diseases, Shahid Beheshti University of Medical Sciences, Tehran, Iran; 2 Department of General Vascular Surgery, Taleghani Hospital, Shahid Beheshti University of Medical Sciences, Tehran, Iran; 3 Department of Microbiology, Pasture Institute of Iran, Tehran, Iran; 4 Department of Medical Bacteriology, Shahid Beheshti University of Med-ical Sciences, Tehran, Iran

**Keywords:** Diabetic foot infection, Antibiotic susceptibility, Multi drug resistance

Dear Editor,

Foot infections are one of the important causes for hospitalization of patients with diabetes and the leading cause of morbidity in diabetic patients.[1][2]

Diabetic foot lesions may present as ulceration, gangrene, charcot joint, or fracture and are associated with amputation if not treated promptly.[3] The proper management of these infections requires early recognition and appropriate antibiotic selection based on culture and antimicrobial susceptibility results and quick initiation of appropriate antibiotic therapy. The aim of current study was to determine the relative frequency of bacterial isolates provided from culture of diabetic foot. We have also included antimicrobial susceptibility tests for commonly used antimicrobial agents to asses the prevalence of antimicrobial resistant patterns within these organisms. During the period of 2007 and 2009, seventy seven diabetic feet infections referring to surgery ward of Taleghani Hospital were included in present study. The samples were ulcer curettages, abscesses and deep tissue needle aspirates. Standard identification tests and antimicrobial susceptibility by disc diffusion method were done on all isolated strains.[4][5]

Staphylococcus aureus, coagulase-negative Staphylococci (CONS), Escherichia coli were the bacterial species most commonly isolated from the patients with diabetic foot lesions (Table 1).

**Table 1:** Bacterial species isolated from patients with diabetic foot infections

***Bacteria* No. (%)**					
*Staphylococcus aureus*	21 (19.4)	*Escherichia coli*	20 (18.4)	*Pseudomonas aeruginosa*	6 (5.6)
*Staphylococcus epidermidis*	20 (18.4)	*Klebsiella spp.*	7 (6.5)	*Acinetobacter spp.*	2 (1.8)
*Other Staphylococci spp.*	4 (3.6)	*Proteus spp.*	5 (4.5)	*Peptostreptococcus spp.*	3 (2.7)
*Enterococci *	7 (6.5)	*Enterobacter spp.*	3 (2.7)	*Peptococcus spp.*	2 (1.8)
*Group D streptococci (Non enterococcus spp)*	3 (2.7)	*Morganella spp.*	1 (0.9)	*Corynebacterium spp.*	3 (2.7)
*Streptococus viridance*	2 (1.8)	Total	109 (100)		

Out of 69 patients with positive cultures, 34 (49%) were only infected with one organism, while others 43 (51%) had mixed infections. The prevalence of aerobeic and anaerobic bacteria were 104 (96.5%) and 5 (4.5 %), respectively. The aerobic and anaerobic organisms both were isolated in diabetic foot ulcers of 4 (6%) patients. S. aureus was the most frequent pathogen (19.4%) that was similar to a previous study in Iran (34.4%).[6]

Antibiotic susceptibility analysis of S. aureus and S. epidermidis exhibited that all of them were methicillin resistant, and majority of the isolates of S. aureus were sensitive to vancomycin and imepenem . Staphylococcus epidermidis was sensitive to vancomycin and imepenem too. High levels of resistance to erythromycin, oxacillin, penicillin, and amoxy/clav were seen among the Enterococcus species. In E. coli, 90%, 95% and 95% of the isolates were resistant to ciprofloxacin, co-Trimoxazole and cephalothin respectively, while 95% of E.coli isolates were sensitive to imepenem. All the isolates of P. aeruginosa were sensitive to imipenem, while clavulanic acid (16%) and ciprofloxacin (16%) showed good activity. All of these isolates were resistant to co-trimoxazole and cephalothin.

The most commonly isolated microorganisms from the diabetic foot lesions in this study were grampositive aerobes, which was in accordance with previous studies in other countries where gram-positive aerobes were the predominant microorganisms isolated from diabetic foot infections.[7][8][9] Other studies from India showed that Proteus species and P. aeruginosa were the most frequently isolated bacteria.[1][9]Polymicrobial infections were shown in 35 (51%) of these infections. The identified anaerobic bacterial isolates belonged to the Peptococcus and Peptostreptococcus genuses which is similar to the findings of Gerding and Smith et al in USA.[10][11] Compared with reports of Abdulrazaka et al. and El-Tahawy (10.5% and 11% respectivly), we have recovered lower rates of anaer obic species (4.5 %).[12][13] Clostridium spp. and Gramnegative anaerobes like Bacteroides spp. and Fusobacterium spp. had been reported in some other studies.[7][14] MRSA had become increasingly prevalent in diabetic foot wounds. All of the isolates of S. aureus were methicillin-resistant, which is in accordance with the report of Ravisekhar et al.[1]

In conclusion, our study showed that gram positive bacterial species are the most frequently isolated bacteria from diabetic foot patients that had multidrug resistance phenotype. Imipenem and vancomycin could be the most effective antimicrobial agents against these bacteria. Further understanding of the causative organisms in diabetic foot infections and their antimicrobial susceptibility pattern are essential for the organization of antimicrobial therapy and management of complications of diabetic foot infections such as foot amputation.
